# Effect of physical activity on the development and the resolution of nonalcoholic fatty liver in relation to body mass index

**DOI:** 10.1186/s12889-022-13128-6

**Published:** 2022-04-05

**Authors:** Hyo-In Choi, Mi Yeon Lee, Hyunah Kim, Byeong Kil Oh, Seung Jae Lee, Jeong Gyu Kang, Sung Ho Lee, Byung Jin Kim, Bum Soo Kim, Jin Ho Kang, Jong-Young Lee, Ki-Chul Sung

**Affiliations:** 1 Division of Cardiology, Department of Internal Medicine, Kangbuk Samsung Hospital, Sungkyunkwan University School of Medicine, 29 Saemunan-ro, Jongno-gu, Seoul, 03181 Republic of Korea; 2 Division of Biostatistics, Department of R&D Management, Kangbuk Samsung Hospital, Sungkyunkwan University School of Medicine, Seoul, Republic of Korea; 3 Center for Cohort Studies, Total Healthcare Center, Kangbuk Samsung Hospital, Sungkyunkwan University School of Medicine, Seoul, Republic of Korea

**Keywords:** Physical activity, Fatty liver, Body-mass index, Prevention, Epidemiology

## Abstract

**Background:**

Data on whether physical activity (PA) levels are related to nonalcoholic fatty liver disease (NAFLD) when considering body mass index (BMI) are scarce. We assessed whether PA affects the development or resolution of NAFLD in conjunction with BMI changes.

**Methods:**

Overall, 130,144 participants who underwent health screening during 2011–2016 were enrolled. According to the PA level in the Korean version of the validated International PA Questionnaire Short Form, participants were classified into the inactive, active, and health-enhancing PA (HEPA) groups.

**Results:**

In participants with increased BMI, the hazard ratio (HR) and 95% confidence interval after multivariable Cox hazard model for incident NAFLD was 0.97 (0.94–1.01) in the active group and 0.94 (0.89–0.99) in the HEPA group, whereas that for NAFLD resolution was 1.03 (0.92–1.16) and 1.04 (0.88–1.23) (reference: inactive group). With increased BMI, high PA affected only new incident NAFLD. PA enhancement or maintenance of sufficient PA prevented new incident NAFLD. In participants with decreased BMI, the HRs were 0.98 (0.90–1.07) and 0.88 (0.78–0.99) for incident NAFLD and 1.07 (0.98–1.17) and 1.33 (1.18–1.49) for NAFLD resolution in the active and HEPA groups, respectively. With decreased BMI, high PA reduced incident NAFLD and increased NAFLD resolution. Maintenance of sufficient PA led to a considerable resolution of NAFLD.

**Conclusion:**

In this large longitudinal study, PA prevented incident NAFLD regardless of BMI changes. For NAFLD resolution, sufficient PA was essential along with BMI decrease. Maintaining sufficient PA or increasing the PA level is crucial for NAFLD prevention or resolution.

**Supplementary Information:**

The online version contains supplementary material available at 10.1186/s12889-022-13128-6.

## Introduction

Nonalcoholic fatty liver disease (NAFLD) is a multisystem disease characterized by fat accumulation in the liver that is not triggered by excessive drinking. Lifestyle and dietary habits have resulted in a considerable increase in the prevalence of NAFLD along with obesity and diabetes [1]. The prevalence of NAFLD as assessed by liver ultrasonography (US) has been reported to range from 17 to 46%, depending on the investigated population [2]. Individuals with NAFLD had a higher overall mortality rate than matched control populations [3], with cardiovascular disease being the most common cause of death [4].

Lifestyle modifications such as diet, exercise, and weight loss have been recommended to improve NAFLD [5]. Most individuals with NAFLD have inadequate physical activity (PA) [6], which is associated with a higher NAFLD risk [7]. We previously reported that moderate exercise was associated with the greatest benefit in terms of preventing NAFLD or improving existing NAFLD, regardless of body mass index (BMI) changes, over a 5-yr follow-up period [8]. However, the impact of PA on the development and resolution of NAFLD in different BMI change groups (increasing or decreasing) could not be determined. Increased PA or exercise frequently results in weight loss, but some individuals gain weight. To the best of our knowledge, no large-scale study has examined the impact of PA on NAFLD in relation to changes in BMI. So, this study aimed to determine whether PA levels are associated with the resolution of existing NAFLD or a reduced risk of incident NAFLD development in different BMI change groups using a large cohort data of Korean adults who underwent regular health examinations.

## Materials and methods

### Study population

Our study population (*n* = 228,589) consisted of individuals who participated at least twice in a comprehensive health screening program that included assessment of PA using the validated Korean version of the International PA Questionnaire Short Form (IPAQ-SF) at Kangbuk Samsung Hospital, Seoul and Suwon, Korea, between 2011 and 2016. The aim of the health screening program was to promote health by detecting chronic diseases and associated risk factors. To examine the association of PA with the incidence or resolution of NAFLD independent of BMI changes, we excluded participants with age < 20 (*n* = 46), missing data on BMI (*n* = 216 at baseline/*n* = 876 at follow-up), waist circumference (n = 38,542 at baseline/*n* = 1116 at follow-up), fatty liver (*n* = 654 at baseline/*n* = 1044 at follow-up), and cancer history (*n* = 5203). Participants were also excluded if they tested positive for hepatitis B surface antigen (HBsAg) or hepatitis C virus (HCV) antibody (*n* = 8090) and if they had a daily alcohol consumption of >20 g (men) or > 10 g (women) (*n* = 49,428). Individuals who were taking medications for hypertension, diabetes, or dyslipidemia were also excluded (*n* = 15,122) to eliminate interference from these factors. Some participants met multiple exclusion criteria, and a total of 130,144 participants were finally included in this study (mean age, 37.16 yr; 52.8% men). The median follow-up duration was 3.03 yr (368,555 person-year). The Kangbuk Samsung Hospital Institutional Review Board approved this study and waived the requirement for informed consent because we used only anonymized and de-identified data obtained as part of the health screening examinations (IRB No. 2018–05-050).

### Data collection and measurements

Information about medical and family history, medication use, lifestyle factors, and education level was obtained through a self-administered questionnaire. Blood pressure and anthropometrical parameters were measured by trained staff during the health examinations. Body weight and height were measured, with the participants in light clothing and without shoes, to the nearest 0.1 kg and 0.1 cm, respectively. BMI was calculated by dividing the weight in kilograms by the height in meters squared. The average amount of alcohol consumed per day was estimated using the frequency and amount of alcohol consumed per drinking day. The PA level was assessed using the validated Korean version of the IPAQ-SF [9]. The IPAQ-SF provides separate scores for walking, moderate-intensity activity, and vigorous-intensity activity, as well as a combined total score to describe the overall level of activity. Additionally, the volume of activity was computed by weighting each type of activity according to its energy requirements defined in metabolic equivalents (MET) to yield a score in MET/min. The participants were categorized according to PA level into the inactive, active, and health-enhancing PA (HEPA) groups [10]. HEPA was defined as any of the following criteria were met: (i) vigorous-intensity activity ≥3 days/wk. accumulating ≥1500 MET min/wk. or (ii) 7 days of any combination of walking, moderate-intensity activity, or vigorous-intensity activity achieving at least 3000 MET min/wk. Participants were characterized as active if any of the following criteria were met: (i) ≥3 days of vigorous-intensity activity for ≥20 min/day, (ii) ≥5 days of moderate-intensity activity or walking for ≥30 min/day, or (iii) ≥5 days of any combination of walking and moderate- or vigorous-intensity activity achieving ≥600 MET min/wk. Participants were characterized as inactive if they did not meet the criteria for either the active or HEPA categories.

Abdominal US (Logic Q700 MR; GE, Milwaukee, WI, USA) was performed by clinical radiologists using a 3.5-MHz probe at baseline and follow-up for all participants. The following images were obtained: (i) sagittal view of the right lobe of the liver and right kidney, (ii) transverse view of the left lateral segment of the liver and spleen, and (iii) transverse view of the liver for altered echo texture. Fatty infiltration of the liver (NAFLD) was considered present if the echogenicity of the liver was greater than that of the renal cortex, with the diaphragm and intrahepatic vessels appearing normal [11]. The inter-observer reliability and intra-observer reliability for fatty liver diagnosis were considered substantial (kappa statistic of 0.74) and excellent (kappa statistic of 0.94), respectively [12]. The Fibrosis-4 (FIB-4) [13] score and NAFLD Fibrosis score [14], indicating the degree of fibrosis, were calculated used baseline measurement data.

### Statistical analyses

Descriptive statistics are used to summarize the characteristics of the participants in separate PA categories. The incidence rate was expressed as the number of cases of incident NAFLD or resolution of NAFLD per 100 person-yr (PY). The adjusted hazard ratio (HR) and 95% CI for incident NAFLD development and NAFLD resolution were estimated using a Cox proportional hazards model. Three regression models were generated for each outcome: model 1 was adjusted for age, sex, center (Seoul or Suwon), year of screening examination, smoking status, alcohol intake, and education level; model 2 was adjusted for variables in model 1 plus waist circumference; and model 3 was adjusted for variables in model 2 plus waist circumference changes. We used the inactive group as the reference category. The Kaplan–Meier curves were used to illustrate time-to-event outcomes in each PA group, with the results being compared using the log rank test. Statistical significance was set at *p* < 0.05. Statistical analyses were conducted using Stata (version 16.1; StataCorp LP, College Station, TX, USA).

## Results

### PA and NAFLD with temporal changes in BMI

A total of 95,959 individuals were identified to have no NAFLD at baseline, of whom 14,066 developed incident NAFLD during the follow-up period. 34,185 participants were identified to have NAFLD at baseline, which resolved in 3755 individuals during the follow-up period. Resolution of NAFLD was defined by absence of US criteria for NAFLD on repeat imaging. Incident NAFLD was defined as NAFLD absence at baseline and presence at follow-up by US. The baseline characteristics of the participants according to NAFLD status are shown in Supporting Table S1. All traditional cardiovascular and metabolic risk factors (age, male sex, lipid profile, fasting glucose, insulin, homeostatic model assessment of insulin resistance [HOMA-IR], blood pressure, and smoking) were more adversely affected in participants with NAFLD than in those without NAFLD. Table 1 shows the baseline characteristics of the participants categorized according to PA levels. Increasing PA categories were positively associated with age, male sex, BMI, systolic blood pressure, alcohol intake, and glucose levels and better insulin resistance markers (lower insulin level and HOMA-IR score). 87,316 individuals increased their BMI during the follow-up period, while 42,828 individuals maintained or decreased their BMI (Table 2). With respect to temporal BMI changes, participants who had increased BMI during the follow-up period had younger age; lower glucose level, insulin level, HOMA-IR score, BMI, and waist circumference; and better lipid profiles at baseline.

**Table 1:** Baseline characteristics according to baseline physical activity groups

	Inactive (*n* = 64,688)	Active (*n *= 46,325)	HEPA (*n* = 19,131)	*P* value
Age (years)	36.71 ± 6.50	37.25 ± 7.09	38.41 ± 8.06	<0.001
Male (n, %)	29,550 (45.99)	27,950 (60.33)	11,002 (57.51)	<0.001
Glucose (mg/dL)	92.76 ± 11.45	93.37 ± 10.93	93.40 ± 10.45	<0.001
AST (IU/L)	20.58 ± 10.44	21.32 ± 10.65	21.94 ± 12.78	<0.001
ALT (IU/L)	21.62 ± 18.44	22.72 ± 18.73	21.41 ± 16.48	<0.001
GGT (IU/L)	26.12 ± 28.55	27.53 ± 27.85	25.45 ± 26.27	<0.001
Triglyceride (mg/dL)^a^	85 (61–125)	88 (63–129)	81 (59–117)	0.0001
HDL-cholesterol (mg/dL)	58.68 ± 14.84	57.87 ± 14.70	59.84 ± 15.15	<0.001
LDL-cholesterol (mg/dL)	117.70 ± 31.42	119.89 ± 31.06	118.61 ± 30.60	<0.001
Insulin (IU/mL)	5.92 ± 3.72	5.66 ± 3.55	5.18 ± 4.01	<0.001
HOMA IR	1.38 ± 0.98	1.33 ± 0.93	1.22 ± 1.02	<0.001
BMI (kg/m^2^)	22.66 ± 3.29	23.08 ± 3.17	23.25 ± 3.05	<0.001
Waist circumference (cm)	80.14 ± 9.48	81.27 ± 9.13	81.02 ± 8.79	<0.001
SBP (mmHg)	105.75 ± 12.37	108.01 ± 12.52	108.85 ± 12.79	<0.001
DBP (mmHg)	68.00 ± 9.48	69.09 ± 9.49	69.02 ± 9.50	0.964
Education				<0.001
≤ High school	8014 (12.39)	3856 (8.32)	3024 (15.81)	
≥ College graduate	50,350 (77.84)	36,438 (78.66)	13,684 (71.53)	
Unknown	6324 (9.78)	6031 (13.02)	2423 (12.67)	
Smoking status (n, %)				<0.001
Never/former smoker	49,091 (75.89)	33,400 (72.10)	13,864 (72.47)	
Current smoker	10,590 (16.37)	8938 (19.29)	3256 (17.02)	
Unknown	5007 (7.74)	3987 (8.61)	2011 (10.51)	
Alcohol (g/day)	4 (1–10)	5 (2–11)	5 (2–11)	0.001
NAFLD	16,447 (25.43)	13,088 (28.25)	4650 (24.31)	<0.001
NAFLD status				<0.001
Incident NAFLD	6316 (9.76)	5390 (11.64)	2360 (12.34)	
Resolution of NAFLD	1825 (2.82)	1404 (3.03)	546 (2.85)	
Temporal BMI changes	0.42 ± 1.22	0.45 ± 1.18	0.52 ± 1.25	<0.001
BMI Change Groups				<0.001
BMI Change >0	21,747 (33.62)	14,995 (32.37)	6086 (31.81)	
BMI Change ≤0	42,941 (66.38)	31,330 (67.63)	13,045 (68.19)	
FIB-4 score	0.73 ± 0.28	0.76 ± 0.31	0.82 ± 0.35	<0.001
NAFLD fibrosis score	−3.07 ± 0.93	−2.99 ± 0.95	−2.83 ± 0.98	<0.001

Numbers are mean (standard deviation), median (interquartile range), or percentages

^a^Triglyceride was log-transformed for this analysis

*Abbreviations*: *BMI* Body mass index, *DBP* Diastolic blood pressure, *FIB-4* Fibrosis-4, *HDL-cholesterol* High-density lipoprotein cholesterol, *HEPA* Health-enhancing physical activity, *HOMA-IR* Homeostatic Model Assessment for Insulin Resistance, *LDL-cholesterol* Low-density lipoprotein cholesterol, *NAFLD* Non-alcoholic fatty liver disease, *SBP* Systolic blood pressure

**Table 2:** Baseline characteristics according to BMI change groups

	BMI Change >0 (*n* = 87,316)	BMI Change ≤0 (*n* = 42,828)	*P* value
Age (years)	36.76 ± 6.74	37.95 ± 7.40	<0.001
Male (n, %)	46,007 (52.69)	22,695 (52.99)	0.307
Glucose (mg/dL)	92.49 ± 10.18	94.26 ± 12.77	<0.001
AST (IU/L)	20.70 ± 10.22	21.76 ± 12.13	<0.001
ALT (IU/L)	21.19 ± 17.37	23.60 ± 19.90	<0.001
GGT (IU/L)	25.50 ± 26.31	28.61 ± 31.02	<0.001
Triglyceride (mg/dL)^a^	83 (60,120)	92 (65,137)	<0.001
HDL-cholesterol (mg/dL)	59.15 ± 14.82	57.35 ± 14.83	<0.001
LDL-cholesterol (mg/dL)	117.26 ± 30.83	121.37 ± 31.74	<0.001
Insulin (IU/mL)	5.52 ± 3.63	6.11 ± 3.84	<0.001
HOMA IR	1.28 ± 0.92	1.46 ± 1.06	<0.001
BMI (kg/m^2^)	22.64 ± 3.18	23.42 ± 3.25	<0.001
Waist circumference (cm)	79.98 ± 9.18	82.08 ± 9.29	<0.001
SBP (mmHg)	106.59 ± 12.39	107.87 ± 12.97	<0.001
DBP (mmHg)	68.27 ± 9.31	69.08 ± 9.85	<0.001
Education			
≤ High school	10,069 (11.53)	4825 (11.27)	
≥ College graduate	67,534 (77.34)	32,938 (76.91)	
Unknown	14,778 (11.36)	5065 (11.83)	
Smoking status (n, %)			<0.001
Never/former smoker	64,505 (73.88)	31,850 (74.37)	
Current smoker	15,585 (17.85)	7199 (16.81)	
Unknown	7226 (8.28)	3779 (8.82)	
Alcohol (g/day)	4 (2, 10)	4 (1, 10)	0.496
NAFLD	20,619 (23.61)	13,566 (31.68)	<0.001
NAFLD status			<0.001
Incident NAFLD	11,654 (13.35)	2412 (5.63)	
Resolution of NAFLD	1336 (1.53)	2439 (5.69)	
Temporal BMI changes	1.04 ± 0.89	−0.77 ± 0.80	<0.001
FIB-4 score	0.74 ± 0.30	0.77 ± 0.32	<0.001
NAFLD fibrosis score	−3.05 ± 0.93	−2.92 ± 0.98	<0.001

Numbers are mean (standard deviation), median (interquartile range), or percentages

^a^Triglyceride was log-transformed for this analysis

*Abbreviations*: *BMI* Body mass index, *DBP* Diastolic blood pressure, *FIB-4* Fibrosis-4, *HDL-cholesterol* High-density lipoprotein cholesterol, *HEPA* Health-enhancing physical activity, *HOMA-IR* Homeostatic Model Assessment for Insulin Resistance, *LDL-cholesterol* Low-density lipoprotein cholesterol, *NAFLD* Non-alcoholic fatty liver disease, *SBP* Systolic blood pressure

**Fig. 1:**
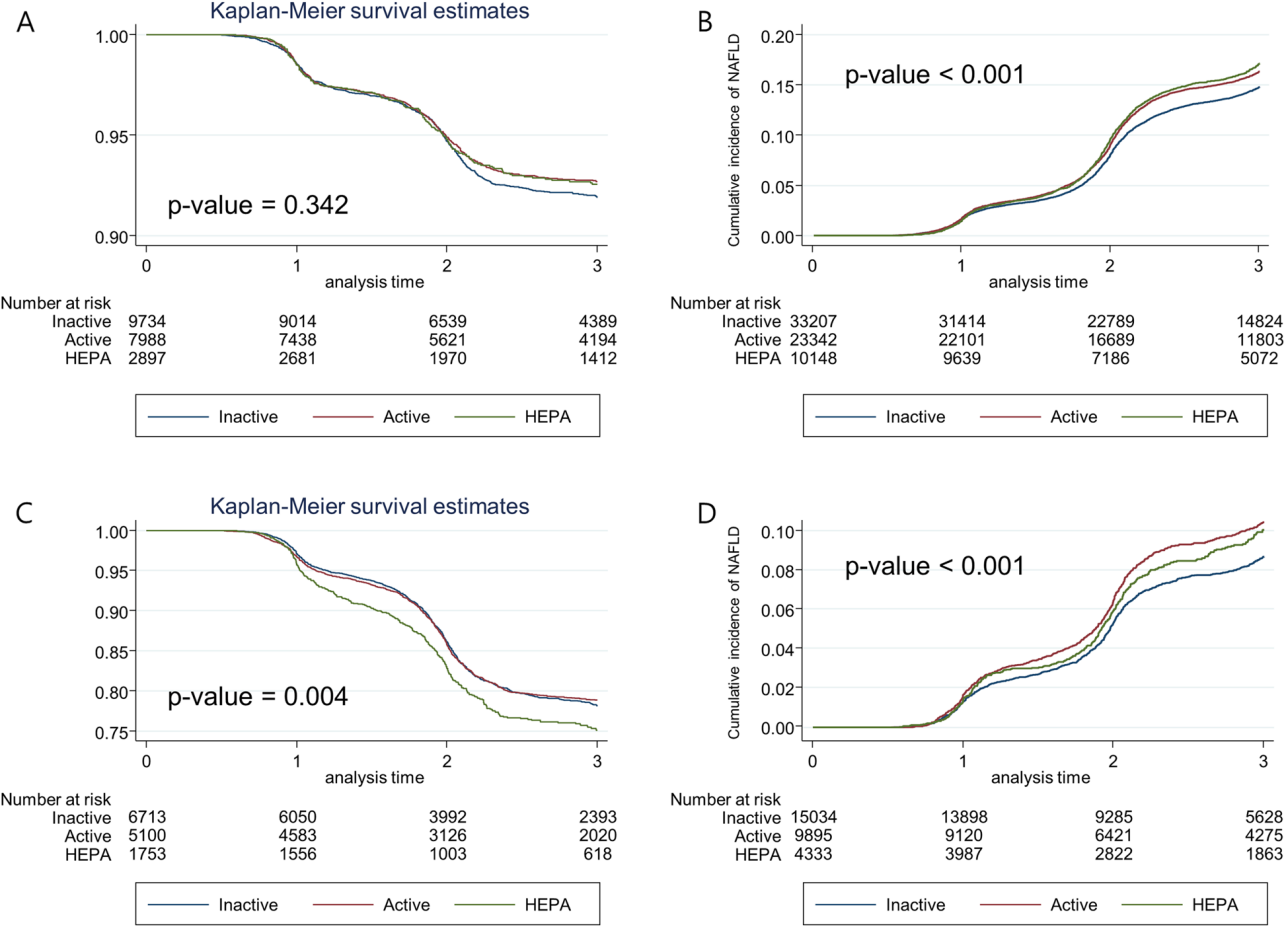
Kaplan-Meier (KM) curves according to physical activity (PA) categories for the resolution and incident development of nonalcoholic fatty liver disease (NAFLD) in different body mass index (BMI) change groups. **A** KM curves for NAFLD resolution in the BMI change >0 group. **B** KM curves for incident NAFLD development in the BMI change >0 group. **C** KM curves for NAFLD resolution in the BMI change ≤0 group. **D** KM curves for incident NAFLD development in the BMI change ≤0 group

The HRs for NAFLD resolution or incident NAFLD development according to the PA category in different BMI change groups are shown in Table 3 (increasing BMI group) and Table 4 (decreasing BMI group). In participants with increased BMI during the follow-up period (Table 3), HEPA was associated with a lower risk of incident NAFLD (HR [95% CI], 0.94 [0.89–0.99], inactive group as reference). However, in increasing BMI group, active PA and HEPA did not show an association with increased resolution of NAFLD (HR [95% CI], 1.03 [0.92–1.16] for active and 1.04 [0.88–1.23] for HEPA group, respectively). In participants with decreased BMI (Table 4), HEPA was associated with a higher chance of NAFLD resolution and a lower risk of incident NAFLD. The multivariable-adjusted HR (95% CI) was 1.33 (1.18–1.49) for resolution of NAFLD and 0.88 (0.78–0.99) for incident NAFLD in HEPA when compared to inactive group as reference. Figure 1 represents event free survival curves for resolution and incidence curve for development of NAFLD in each BMI change groups. For the resolution of NAFLD, the decreased BMI group showed statistically significant differences between PA groups, and for the incidence of NAFLD, both BMI change groups showed statistically significant differences between PA groups. Supporting Fig. S1 shows spline curves displaying the risk of (A) resolution and (B) incidence of NAFLD in overall and the three activity groups (Model 3), which shows the graphical pattern of association.

**Table 3:** HR of resolution and incident NAFLD according to PA category in BMI change >0 group

	Person-years	Events (No.)	Event rate (per 100,000 person-years)	Age- and sex-adjusted HR (95% CI)	Multivariable-adjusted HR (95% CI)		
					Model 1	Model 2	Model 3
Resolution of NAFLD							
Inactive	27,572.3	644	2.3	1.00	1.00	1.00	1.00
Active	24,221.6	507	2.1	1.00 (0.89–1.12)	1.04 (0.92–1.17)	1.02 (0.91–1.15)	1.03 (0.92–1.16)
HEPA	8493.0	185	2.2	0.96 (0.82–1.14)	1.01 (0.86–1.19)	1.01 (0.85–1.19)	1.04 (0.88–1.23)
Incident NAFLD							
Inactive	94,031.3	5216	5.5	1.00	1.00	1.00	1.00
Active	69,460.8	4455	6.4	0.95 (1.91–0.99)	0.98 (0.94–1.02)	0.98 (0.94–1.02)	0.97 (0.94–1.01)
HEPA	30,079.8	1983	6.6	0.97 (0.92–1.02)	0.99 (0.94–1.05)	0.99 (0.94–1.04)	0.94 (0.89–0.99)

Model 1: adjustment for age, sex, center, year of screening exam, smoking status, alcohol intake, education level

Model 2: model 1 adjustments plus adjustment for waist circumference

Model 3: model 2 adjustments plus adjustment for waist circumference changes

The reference group was inactive group

*Abbreviations*: *BMI* Body mass index, *CI* Confidence interval, *HEPA* Health-enhancing physical activity, *HR* Hazard ratio, *NAFLD* Non-alcoholic fatty liver disease, *PA* Physical activity

**Table 4:** HR of resolution and incident NAFLD according to PA category in BMI change ≤0 group

	Person-years	Events (No.)	Event rate (per 100,000 person-years)	Age- and sex-adjusted HR (95% CI)	Multivariable-adjusted HR (95% CI)		
					Model 1	Model 2	Model 3
Resolution of NAFLD							
Inactive	17,358.3	1181	6.8	1.00	1.00	1.00	1.00
Active	13,624.5	897	6.6	1.05 (0.97–1.15)	1.10 (1.01–1.20)	1.06 (0.97–1.16)	1.07 (0.98–1.17)
HEPA	4504.8	361	8.0	1.23 (1.09–1.38)	1.33 (1.19–1.50)	1.31 (1.16–1.47)	1.33 (1.18–1.49)
Incident NAFLD							
Inactive	39,646.9	1100	2.8	1.00	1.00	1.00	1.00
Active	27,526.6	935	3.4	0.99 (0.91–1.08)	0.97 (0.89–1.06)	0.99 (0.91–1.09)	0.98 (0.90–1.07)
HEPA	12,035.9	377	3.1	0.86 (0.76–0.96)	0.85 (0.76–0.96)	0.88 (0.78–0.99)	0.88 (0.78–0.99)

Model 1: adjustment for age, sex, center, year of screening exam, smoking status, alcohol intake, education level

Model 2: model 1 adjustments plus adjustment for waist circumference

Model 3: model 2 adjustments plus adjustment for waist circumference changes

The reference group was inactive group

*Abbreviations*: *BMI* Body mass index, *CI* Confidence interval, *HEPA* Health-enhancing physical activity, *HR* Hazard ratio, *NAFLD* Non-alcoholic fatty liver disease, *PA* Physical activity

We divided the study participants into three groups to examine if there was a difference in the influence on the resolution or development of NAFLD according to their initial BMI: underweight (BMI < 18.5), normal (BMI 18.5–23), and overweight (BMI > 23) (Supporting Table S2). For the risk of incident NAFLD, overweight individuals showed greatest benefit with active PA or HEPA in both BMI change groups. In decreasing BMI group, overweight NAFLD individuals showed greatest benefits in resolution of NAFLD with active PA or HEPA. NAFLD individuals with normal BMI benefits with active PA or HEPA even in case of increasing BMI during follow-up.

### Temporal changes in PA and NAFLD with temporal changes in BMI

We found an association of temporal changes in PA with the resolution and development of NAFLD in participants with temporal BMI changes (Tables 5 and 6). PA maintenance or enhancement was associated with a lower risk of incident NAFLD despite an increase in BMI (Table 5; HR 0.90 [0.85–0.96] for the inactive to active/HEPA group and HR 0.89 [0.85–0.93] for the active/HEPA to active/HEPA group); however, PA maintenance or enhancement was not associated with the resolution of NAFLD if the BMI increased (HR 1.16 [0.99–1.36] for the inactive to active/HEPA group and HR 1.10 [0.96–1.27] for the active/HEPA to active/HEPA group). Among participants with decreased BMI (Table 6), those with persistent active PA or HEPA showed increased resolution of NAFLD (HR 1.18 [1.07–1.31]). In this subset, the HR for incidence of NAFLD was 0.99 (0.89–1.12) for the PA enhancement category and 0.93 (0.83–1.03) for the PA maintenance category.

**Table 5:** HR of resolution and incident NAFLD according to temporal PA change in BMI change >0 group

	Person-years	Events (No.)	Event rate (per 100,000 person-years)	Age- and sex-adjusted HR (95% CI)	Multivariable-adjusted HR (95% CI)		
					Model 1	Model 2	Model 3
Resolution of NAFLD							
Inactive→Inactive	18,027.0	407	2.25	1.00	1.00	1.00	1.00
Active or HEPA→Inactive	12,962.8	268	2.06	0.98 (0.84–1.15)	1.04 (0.89–1.22)	1.04 (0.89–1.21)	1.07 (0.91–1.25)
Inactive- → Active or HEPA	9545.3	237	2.48	1.17 (0.99–1.37)	1.17 (0.99–1.37)	1.19 (1.02–1.40)	1.16 (0.99–1.36)
Active or HEPA→Active or HEPA	19,751.9	424	2.14	1.08 (0.94–1.24)	1.12 (0.98–1.29)	1.11 (0.97–1.28)	1.10 (0.96–1.27)
Incident NAFLD							
Inactive→Inactive	63,556.4	3448	5.45	1.00	1.00	1.00	1.00
Active or HEPA→Inactive	40,517.9	2617	6.45	0.99 (0.94–1.05)	1.04 (0.98–1.09)	1.03 (0.98–1.08)	0.99 (0.94–1.04)
Inactive- → Active or HEPA	30,474.9	1768	5.80	0.90 (0.85–0.96)	0.91 (0.86–0.96)	0.89 (0.84–0.94)	0.90 (0.85–0.96)
Active or HEPA→Active or HEPA	59,022.7	3821	6.47	0.87 (0.83–0.92)	0.90 (0.86–0.94)	0.89 (0.85–0.94)	0.89 (0.85–0.93)

Model 1: adjustment for age, sex, center, year of screening exam, smoking status, alcohol intake, education level

Model 2: model 1 adjustments plus adjustment for waist circumference

Model 3: model 2 adjustments plus adjustment for waist circumference changes

The reference group was persistently inactive group

*Abbreviations*: *BMI* Body mass index, *CI* Confidence interval, *HEPA* Health-enhancing physical activity, *HR* Hazard ratio, *NAFLD* Non-alcoholic fatty liver disease, *PA* Physical activity

**Table 6:** HR of resolution and incident NAFLD according to temporal PA change in BMI change ≤0 group

	Person-years	Events (No.)	Event rate (per 100,000 person-years)	Age- and sex-adjusted HR (95% CI)	Multivariable-adjusted HR (95% CI)		
					Model 1	Model 2	Model 3
Resolution of NAFLD							
Inactive→Inactive	9984.5	679	6.80	1.00	1.00	1.00	1.00
Active or HEPA→Inactive	6053.5	381	6.29	0.97 (0.86–1.10)	1.04 (0.92–1.18)	1.01 (0.89–1.15)	1.04 (0.92–1.18)
Inactive- → Active or HEPA	7373.8	502	6.80	1.05 (0.94–1.18)	1.05 (0.94–1.18)	1.08 (0.97–1.22)	1.00 (0.89–1.13)
Active or HEPA→Active or HEPA	12,075.7	877	7.26	1.21 (1.09–1.34)	1.26 (1.14–1.40)	1.24 (1.12–1.38)	1.18 (1.07–1.31)
Incident NAFLD							
Inactive→Inactive	25,070.9	650	2.59	1.00	1.00	1.00	1.00
Active or HEPA→Inactive	14,209.1	452	3.18	1.04 (0.92–1.17)	1.02 (0.91–1.15)	1.02 (0.90–1.15)	1.00 (0.89–1.13)
Inactive- → Active or HEPA	14,575.9	450	3.08	1.03 (0.91–1.16)	1.03 (0.91–1.16)	0.94 (0.83–1.06)	0.99 (0.88–1.12)
Active or HEPA→Active or HEPA	25,353.3	860	3.39	0.92 (0.83–1.02)	0.90 (0.82–1.00)	0.89 (0.80–0.99)	0.93 (0.83–1.03)

Model 1: adjustment for age, sex, center, year of screening exam, smoking status, alcohol intake, education level

Model 2: model 1 adjustments plus adjustment for waist circumference

Model 3: model 2 adjustments plus adjustment for waist circumference changes

The reference group was persistently inactive group

*Abbreviations*: *BMI* Body mass index, *CI* Confidence interval, *HEPA* Health-enhancing physical activity, *HR* Hazard ratio, *NAFLD* Non-alcoholic fatty liver disease, *PA* Physical activity

## Discussion

This large longitudinal study identified several insightful findings. First, HEPA prevented incident NAFLD regardless of BMI changes. Second, HEPA is also related to the resolution of existing NAFLD when combined with BMI reduction. Third, PA maintenance or enhancement over the 3-yr follow-up period was related to the resolution of NAFLD and a decreased risk of incident NAFLD. These findings suggest that increasing PA could be effective strategy to prevent or resolute NAFLD even in the absence of BMI reduction.

The current NAFLD management includes dietary and PA modifications mainly aimed at weight loss [5]. Previous studies reported that exercise which did not induce weight loss can improve hepatic steatosis [15, 16]. Another study involving both obese and lean adolescents showed that a 12-wk aerobic exercise program reduced hepatic fat accumulation and insulin resistance in the absence of weight loss [15]. Even in the absence of weight reduction, short-term aerobic exercise training reduced the hepatic lipid content, as assessed using magnetic resonance imaging and proton magnetic resonance spectroscopy (^1^H-MRS) [16]. A small randomized trial demonstrated that resistance exercise also reduced hepatic fat, increased insulin sensitivity, and improved metabolic flexibility in participants with NAFLD, independent of weight loss [17]. These studies suggest that reduction in liver fat content is possible with exercise even in the absence of a considerable change in body weight.

In our longitudinal cohort study, a higher level of PA at baseline was associated with an increase in NAFLD resolution and a decrease in subsequent NAFLD development, even after adjustment for visceral obesity (waist circumference). In our previous study, which included 36,195 US-diagnosed NAFLD patients, 19.6% had their NAFLD resolved after a mean follow-up of 4.9 ± 3.4 years [18]. In a Swedish cohort study, 129 individuals with biopsy-proven NAFLD were followed for 13.7 ± 1.3 years, and reported changes in fibrosis stage between baseline and follow-up [19]. During the 3.03-year follow-up period in present study, 3775 of 30,410 NAFLD patients resolved NAFLD, while 14,066 of 95,959 without NAFLD developed NAFLD. Despite the relatively short follow-up duration, these findings are consistent with those of previous studies. The impact of increased PA was more pronounced when combined with BMI reduction. Despite worse baseline metabolic risk profiles than those with inactive PA, the HEPA group had a reduced incidence of NAFLD. This further emphasizes the importance of PA which might overcome worse metabolic risk profiles. The analysis according to the baseline BMI subgroups suggests that the overweight individual can benefit more from active PA or HEPA (Supporting Table S2). Systematic review showed that exercise only interventions without weight loss produce a modest but significant effect upon liver lipid (^1^H-MRS measured intrahepatic triacyglycerol concentration (IHTAG) of 1.8%, relative reduction of 21%) and lifestyle interventions producing weight loss significantly improve liver lipid (absolute reductions in IHTAG of 2–4.6%, relative reductions of 13–51%) [20]. These results could be interpreted that PA has beneficial effects regardless of weight changes, but the beneficial effects are more pronounced when weight loss is achieved, especially for the resolution of existing NAFLD. Abolition of the benefits of PA by weight gain may be a direct effect of weight gain itself or may be explained by the pathologic processes that cause increased body weight; however, the exact mechanisms warrant further studies. Since the prevalence or incidence of NAFLD in the underweight group was very low (Supporting Table S2), it is difficult to draw a clear conclusion on these cases (whether weight gain or loss has association with NAFLD in underweight NAFLD individuals), and further studies are needed in these population. Our findings emphasize the importance of PA in the resolution and prevention of NAFLD, as well as the greater benefits could be achieved when combined with a decrease in BMI. Furthermore, the amount of PA was associated with the resolution or prevention of NAFLD in a dose-dependent manner. Our findings are also in accordance with the current guidelines for increasing PA and adopting long-term lifestyle changes [21].

With respect to temporal changes in PA, our study showed that increasing the amount of PA to an active status or maintaining an active PA or HEPA status during the median follow-up of 3 yr was associated with NAFLD resolution or prevention. Compared with the persistently inactive group, active PA or HEPA at follow-up demonstrated a higher benefit on NAFLD resolution than baseline PA level. A cross-sectional study conducted in Korea showed that an increasingly sedentary lifestyle was associated with a high prevalence of NAFLD and that the risk of developing NAFLD decreased by 6% in the minimally active group compared with the inactive group [22]. Given the paucity of evidence, our results suggest that even a relatively short period of PA enhancement may provide a benefit to individuals with NAFLD. PA is thought to improve fatty liver through a variety of mechanisms. In previous studies, PA have been shown to reduce hepatic fat content through improvements in insulin resistance [23], liver fatty acid metabolism [24], liver mitochondrial function, and activation of inflammatory cascades [25].

Our study had some limitations. An important limitation of this study was that the onset of new NAFLD and changes in PA levels could not be identified. Additional large-scale prospective studies are required to overcome this limitation. Present study used self-reported PA questionnaire which may be inaccurate. However, the IPAQ-SF is a widely used questionnaire in research that can determine the amount and frequency of PA relatively accurately. We diagnosed NAFLD using abdominal US. Liver biopsy is the gold standard method for the quantitative diagnosis of NAFLD. Nevertheless, US is currently the preferred method for the initial screening of NAFLD. Moreover, the sensitivity and specificity of US in diagnosing moderate to severe steatosis are rather high (78.4–90.8% and 76.0–90.0%, respectively) [26]. We used as much information as possible to exclude patients with underlying liver disease, but due to the limitations of the large database established by the health examination program, patients with liver diseases such as autoimmune liver disease or cholestatic disease may not be considered. However, since this cohort consisted of relatively young and healthy individuals, the risk of data contamination due to the aforementioned factors is not expected to be high. Also, we were unable to account for other concurrent variables such as dietary changes, dietary content, and medications used. Despite the limitations, the novelty of the study design and the large number of included participants are the strengths of our study.

## Conclusion

In this large longitudinal study, PA was found to be associated with the prevention of NAFLD regardless of BMI changes. PA can result in the resolution of existing NAFLD particularly when combined with BMI reduction. Maintenance or enhancement of PA even for a short period can provide benefits in terms of NAFLD prevention or resolution.

## Supplementary Information

**Additional file 1: Supporting Fig. S1.** Spline curves displaying the risk of (A) resolution and (B) incidence of NAFLD in overall and the three activity groups (Model 3).

**Additional file 2: Supporting Table S1.** Baseline Characteristics of the Cohort Stratified Baseline NAFLD Status. **Supporting Table S2.** HR of Resolution and Incident NAFLD According to PA in BMI Change >0 Group (Splited by BMI Category at Baseline).

## Data Availability

Data are available upon request from the corresponding author due to institutional data protection. Research results must be reviewed through the corresponding author according to the guidelines for research results management of Korea Centers for Disease Control and Prevention. The interested researchers may contact to the corresponding author, Dr. Jong-Young Lee, e-mail address: jyleeheart@naver.com. Although the data are not available to be shared publicly, data are provided directly from the corresponding author to the individual researchers.

## Abbreviations

ALT: Alanine aminotransferase
AST: Aspartate aminotransferase
BMI: Body mass index
DBP: Diastolic blood pressure
GGT: Gamma-glutamyl transferase
HEPA: Health-enhancing physical activity
HOMA-IR: Homeostatic model assessment of insulin resistance
HR: Hazard ratio
IPAQ-SF: International physical activity questionnaire short form
MET: Metabolic equivalents
NAFLD: Nonalcoholic fatty liver disease
PA: Physical activity
SBP: Systolic blood pressure
US: Ultrasonography
