# Supplementary material for: Effect of physical activity on the development and the resolution of nonalcoholic fatty liver in relation to body mass index
Source: BMC Public Health. 2022 Apr 5;22:655. doi: 10.1186/s12889-022-13128-6 (PMC8985384; doi:10.1186/s12889-022-13128-6)
Supplement: Supplementary file 1 — Additional file 1: Supporting Fig. S1. Spline curves displaying the risk of (A) resolution and (B) incidence of NAFLD in overall and the three activity groups (Model 3). [file 12889_2022_13128_MOESM1_ESM.zip › Supporting figure legend.docx]

Supporting figure legend

SUPPORTING FIG. S1. Spline curves displaying the risk of (A) resolution and (B) incidence of NAFLD in overall and the three activity groups (Model 3).
