# Supplementary material for: Effect of physical activity on the development and the resolution of nonalcoholic fatty liver in relation to body mass index
Source: BMC Public Health. 2022 Apr 5;22:655. doi: 10.1186/s12889-022-13128-6 (PMC8985384; doi:10.1186/s12889-022-13128-6)
Supplement: Supplementary file 2 — Additional file 2: Supporting Table S1. Baseline Characteristics of the Cohort Stratified Baseline NAFLD Status. Supporting Table S2. HR of Resolution and Incident NAFLD According to PA in BMI Change >0 Group (Splited by BMI Category at Baseline). [file 12889_2022_13128_MOESM2_ESM.docx]

**Supporting Tables**

| **SUPPORTING TABLE S1. Baseline Characteristics of the Cohort Stratified Baseline NAFLD Status** | | | |
| --- | --- | --- | --- |
|  | NAFLD (n=34,185) | No NAFLD (n=95,959) | *P* value |
| Age (years) | 38.66±7.09 | 36.61±6.87 | <0.001 |
| Male (n, %) | 28,082 (82.15) | 40,620 (42.33) | <0.001 |
| Glucose (mg/dL) | 97.65±15.27 | 91.44±8.65 | <0.001 |
| AST (IU/L) | 25.73±13.47 | 19.38±9.27 | <0.001 |
| ALT (IU/L) | 34.51±26.20 | 17.19±11.02 | <0.001 |
| GGT (IU/L) | 42.23±37.24 | 20.93±21.19 | <0.001 |
| Triglyceride (mg/dL)* | 133 (96-185) | 75(57-02) | <0.001 |
| HDL-cholesterol (mg/dL) | 48.78±11.10 | 62.04±14.45 | <0.001 |
| LDL-cholesterol (mg/dL) | 135.57±31.02 | 112.57±28.94 | <0.001 |
| Insulin (IU/mL) | 8.02±4.53 | 4.89±2.97 | <0.001 |
| HOMA IR | 1.96±1.26 | 1.12±0.72 | <0.001 |
| BMI (kg/m^2^) | 25.82±2.95 | 21.85±2.62 | <0.001 |
| Waist circumference (cm) | 89.12±7.60 | 77.66±.84 | <0.001 |
| SBP (mmHg) | 114.18±12.11 | 104.46±11.70 | <0.001 |
| DBP (mmHg) | 73.48±9.62 | 66.78±8.18 | <0.001 |
| Education |  |  | <0.001 |
| ≤High school | 3,615 (10.57) | 11,279 (11.75) |  |
| ≥College graduate | 26,517 (77.57) | 73,955 (77.07) |  |
| Unknown | 4,053 (11.86) | 10,725 (11.18) |  |
| Smoking status (n, %) |  |  | <0.001 |
| Never/former smoker | 22,339 (65.35) | 74,016 (77.13) |  |
| Current smoker | 9,638 (28.19) | 13,146 (13.70) |  |
| Unknown | 2,208 (6.46) | 8,797 (9.17) |  |
| Alcohol (g/day) | 7 (3-14) | 4 (1-10) | <0.001 |
| BMI change |  |  | <0.001 |
| >0 | 13,566 (39.68) | 29,262 (30.49) |  |
| ≤0 | 20,619 (60.32) | 66,697 (69.51) |  |
| FIB-4 | 0.72±0.31 | 0.76±0.31 | <0.001 |
| NAFLD fibrosis score | -2.93±1.0 | -3.03±0.93 | <0.001 |
| Numbers are mean (standard deviation), median (interquartile range), or percentages.  ^*^Triglyceride was log-transformed for this analysis.  Abbreviations: BMI, body mass index; DBP, diastolic blood pressure; FIB-4 index; fibrosis-4 index, HDL-cholesterol, high-density lipoprotein cholesterol; HEPA, health-enhancing physical activity; HOMA-IR, homeostatic model assessment for insulin resistance; LDL-cholesterol, low-density lipoprotein cholesterol; NAFLD, non-alcoholic fatty liver disease; SBP, systolic blood pressure. | | | |

| **SUPPORTING TABLE S2. HR of Resolution and Incident NAFLD According to PA in BMI Change >0 Group (Splited by BMI Category at Baseline)** | | | | | | | |
| --- | --- | --- | --- | --- | --- | --- | --- |
|  | Person-years | Events (No.) | Incident rate (per 100 person-years) | Age- and sex-adjusted HR (95% CI) | Multivariable-adjusted HR (95% CI) | | |
|  |  |  |  |  | Model 1 | Model 2 | Model 3 |
| Resolution of NAFLD |  |  |  |  |  |  |  |
| Inactive | 27,572.3 | 644 | 2.3 | 1.00 | 1.00 | 1.00 | 1.00 |
| Active | 24,221.6 | 507 | 2.1 | 1.00 (0.89-1.12) | 1.04 (0.92-1.17) | 1.02 (0.91-1.15) | 1.03 (0.92-1.16) |
| HEPA | 8,493.0 | 185 | 2.2 | 0.96 (0.82-1.14) | 1.01 (0.86-1.19) | 1.01 (0.85-1.19) | 1.04 (0.88-1.23) |
| *P* for trend |  |  |  | 0.703 | 0.735 | 0.832 | 0.567 |
| Underweight (BMI<18.5) |  |  |  |  |  |  |  |
| Inactive | 33.23 | 7 | 21.06 | 1 (reference) | 1 (reference) | 1 (reference) | 1 (reference) |
| Active | 16.28 | 4 | 24.57 | 0.86 (0.21-3.48) | 0.8 (0.07-9.56) | 0.49 (0.03-8.88) | 1.93 (0.08-46.5) |
| HEPA | 5.68 | 2 | 35.24 | 1.53 (0.3-7.69) | 4.55 (0.07-283.64) | 188.16 (0.23-156000) | 25.01 (0.21 - 3030) |
| *P* for trend |  |  |  | 0.746 | 0.641 | 0.561 | 0.193 |
| Normal (18.5-23) |  |  |  |  |  |  |  |
| Inactive | 4,722.04 | 240 | 5.08 | 1 (reference) | 1 (reference) | 1 (reference) | 1 (reference) |
| Active | 3,715.44 | 202 | 5.44 | 1.21 (1.01-1.47) | 1.28 (1.06-1.54) | 1.22 (1.01-1.48) | 1.23 (1.02-1.49) |
| HEPA | 1,107.77 | 65 | 5.87 | 1.21 (0.92-1.59) | 1.27 (0.96-1.68) | 1.21 (0.92-1.6) | 1.21 (0.92 - 1.6) |
| *P* for trend |  |  |  | 0.055 | 0.016 | 0.053 | 0.051 |
| Overweight (≥23) |  |  |  |  |  |  |  |
| Inactive | 22,817.05 | 397 | 1.74 | 1 (reference) | 1 (reference) | 1 (reference) | 1 (reference) |
| Active | 20,489.94 | 301 | 1.47 | 0.9 (0.78-1.05) | 0.95 (0.82-1.11) | 0.94 (0.81-1.09) | 0.94 (0.81-1.1) |
| HEPA | 7,379.64 | 118 | 1.6 | 0.93 (0.75-1.14) | 0.97 (0.79-1.2) | 0.94 (0.77-1.16) | 0.98 (0.8-1.21) |
| *P* for trend |  |  |  | 0.278 | 0.660 | 0.452 | 0.654 |
| *P* for interaction |  |  |  | 0.218 | 0.186 | 0.206 | 0.162 |
| Incident NAFLD |  |  |  |  |  |  |  |
| Inactive | 94,031.3 | 5,216 | 5.5 | 1.00 | 1.00 | 1.00 | 1.00 |
| Active | 69,460.8 | 4,455 | 6.4 | 0.95 (1.91-0.99) | 0.98 (0.94-1.02) | 0.98 (0.94-1.02) | 0.97 (0.94-1.01) |
| HEPA | 30,079.8 | 1,983 | 6.6 | 0.97 (0.92-1.02) | 0.99 (0.94-1.05) | 0.99 (0.94-1.04) | 0.94 (0.89-0.99) |
| *P* for trend |  |  |  | 0.066 | 0.631 | 0.537 | 0.022 |
| Underweight (BMI<18.5) |  |  |  |  |  |  |  |
| Inactive | 11,865.79 | 68 | 0.57 | 1 (reference) | 1 (reference) | 1 (reference) | 1 (reference) |
| Active | 6,438.58 | 39 | 0.61 | 0.85 (0.57-1.27) | 0.9 (0.61-1.35) | 0.92 (0.62-1.37) | 0.91 (0.61-1.36) |
| HEPA | 1,912.93 | 10 | 0.52 | 0.81 (0.42-1.58) | 0.83 (0.43-1.63) | 0.85 (0.43-1.66) | 0.9 (0.46-1.76) |
| *P* for trend |  |  |  | 0.386 | 0.511 | 0.565 | 0.631 |
| Normal (18.5-23) |  |  |  |  |  |  |  |
| Inactive | 59,684.42 | 2,124 | 3.56 | 1 (reference) | 1 (reference) | 1 (reference) | 1 (reference) |
| Active | 42,617.51 | 1,695 | 3.98 | 0.93 (0.88-0.996) | 0.96 (0.9-1.02) | 0.97 (0.91-1.03) | 0.97 (0.91-1.03) |
| HEPA | 17,937.15 | 669 | 3.73 | 0.89 (0.81-0.97) | 0.92 (0.84-1.001) | 0.97 (0.89-1.06) | 0.94 (0.86-1.02) |
| *P* for trend |  |  |  | 0.004 | 0.037 | 0.353 | 0.118 |
| Overweight (≥23) |  |  |  |  |  |  |  |
| Inactive | 22,481.1 | 3,024 | 13.45 | 1 (reference) | 1 (reference) | 1 (reference) | 1 (reference) |
| Active | 20,404.71 | 2,721 | 13.34 | 0.92 (0.88-0.97) | 0.95 (0.9-1.01) | 0.97 (0.92-1.03) | 0.97 (0.92-1.02) |
| HEPA | 10,229.72 | 1,304 | 12.75 | 0.88 (0.83-0.94) | 0.91 (0.85-0.97) | 0.95 (0.89-1.02) | 0.92 (0.86-0.98) |
| *P* for trend |  |  |  | <0.001 | 0.002 | 0.127 | 0.010 |
| *P* for interaction |  |  |  | 0.278 | 0.309 | 0.842 | 0.881 |
| Model 1: adjustment for age, sex, center, year of screening exam, smoking status, alcohol intake, education level  Model 2: model 1 adjustments plus adjustment for waist circumference  Model 3: model 2 adjustments plus adjustment for waist circumference changes  The reference group was inactive group.  Abbreviations: BMI, body mass index; CI, confidence interval; HEPA, health-enhancing physical activity; HR, hazard ratio; NAFLD, non-alcoholic fatty liver disease; PA, physical activity. | | | | | | | |
